# Language, Gesture, and Emotional Communication: An Embodied View of Social Interaction

**DOI:** 10.3389/fpsyg.2019.02063

**Published:** 2019-09-24

**Authors:** Elisa De Stefani, Doriana De Marco

**Affiliations:** ^1^Dipartimento di Neuroscienze, Università di Parma, Parma, Italy; ^2^Consiglio Nazionale delle Ricerche, Istituto di Neuroscienze, Parma, Italy

**Keywords:** gesture, language, embodied cognition, mirror neurons, emotional communication, abstract concepts, motor resonance, social interaction

## Abstract

Spoken language is an innate ability of the human being and represents the most widespread mode of social communication. The ability to share concepts, intentions and feelings, and also to respond to what others are feeling/saying is crucial during social interactions. A growing body of evidence suggests that language evolved from manual gestures, gradually incorporating motor acts with vocal elements. In this evolutionary context, the human mirror mechanism (MM) would permit the passage from “doing something” to “communicating it to someone else.” In this perspective, the MM would mediate semantic processes being involved in both the execution and in the understanding of messages expressed by words or gestures. Thus, the recognition of action related words would activate somatosensory regions, reflecting the semantic grounding of these symbols in action information. Here, the role of the sensorimotor cortex and in general of the human MM on both language perception and understanding is addressed, focusing on recent studies on the integration between symbolic gestures and speech. We conclude documenting some evidence about MM in coding also the emotional aspects conveyed by manual, facial and body signals during communication, and how they act in concert with language to modulate other’s message comprehension and behavior, in line with an “embodied” and integrated view of social interaction.

## Introduction

In the last years, the hypothesis of language as “embodied” in sensory and motor experience has been widely discussed in the field cognitive neuroscience.

In this review, we will firstly discuss recent behavioral and neurophysiological studies confirming the essential role of sensorimotor brain areas in language processing, facing the controversial issues and reviewing recent results that suggest an extended view of embodied theories.

We will discuss this hypothesis, providing evidences about the gestural origin of language, focusing on studies investigating the functional relation between manual gesture and speech and the neural circuits involved in their processing and production.

Finally, we will report evidences about the functional role of manual and facial gestures as communicative signals that, in concert with language, express emotional messages in the extended context of social interaction.

All these points provide evidences in favor of an integrated body/verbal communication system mediated by the mirror mechanism (MM).

## What Is Embodied About Communication? the Involvement of Mirror Mechanism in Language Processing

It is well known that our thoughts are verbally expressed by symbols that have little or no physical relationship with objects, actions and feelings to which they refer. Knowing how linguistic symbols may have been associated with aspects of the real world represents one of the thorniest issues about the study of language and its evolution. In cognitive psychology, a classic debate has concerned how language is stored and recovered in the human brain.

According to the classical “amodal approach,” the concepts are expressed in a symbolic format (Fodor, 1998; Mahon and Caramazza, 2009). The core assumption is that meanings of words are like a formal language, composed of arbitrary symbols, which represent aspects of the word (Chomsky, 1980; Kintsch, 1998; Fodor, 2000); to understand a sentence, words are led back symbols that represent their meaning. In other terms, there would be an arbitrary relationship between the word and its referent (Fodor, 1975, 2000; Pinker, 1994; Burgess and Lund, 1997; Kintsch, 1998). Neuropsychological studies provide interesting evidence for the amodal nature of concept. In Semantic Dementia, for example, a brain damage in the temporal and adjacent areas results in an impairment of conceptual processing (Patterson et al., 2007). A characteristic of this form of dementia is the degeneration of the anterior temporal lobe (ATL) that several imaging studies have highlighted to have a critical role in amodal conceptual representations (for a meta-analysis, see Visser et al., 2010).

In contrast, the embodied approaches to language propose that conceptual knowledge is grounded in body experience and in the sensorimotor systems (Gallese and Lakoff, 2005; Barsalou, 2008; Casile, 2012) that are involved in forming and retrieving semantic knowledge (Kiefer and Pulvermüller, 2012). These theories are supported by the discovery of mirror neurons (MNs), identified in the ventral pre-motor area (F5) of the macaque (Gallese et al., 1996; Rizzolatti et al., 2014). MNs would be at the basis of both action comprehension and language understanding, constituting the neural substrate from which more sophisticated forms of communication evolved (Rizzolatti and Arbib, 1998; Corballis, 2010). The MM is based on the process of motor resonance, which mediates action comprehension: when we observe someone performing an action, the visual input of the observed motor act reaches and activates the same fronto-parietal networks recruited during the execution of the same action (Nelissen et al., 2011), permitting a direct access to the own motor representation. This mechanism was hypothesized to be extended to language comprehension, namely when we listen a word or a sentence related to an action (e.g., “grasping an apple”), allowing an automatic access to action/word semantics (Glenberg and Kaschak, 2002; Pulvermüller, 2005; Fischer and Zwaan, 2008; Innocenti et al., 2014; Vukovic et al., 2017; Courson et al., 2018; Dalla Volta et al., 2018). This means that we comprehend words referring to concrete objects or actions directly accessing to their meaning through our sensorimotor experience (Barsalou, 2008).

The sensorimotor activation in response to language processing was demonstrated by a large amount of neurophysiological studies. Functional magnetic resonance imaging (fMRI) studies demonstrated that seeing action verbs activated similar motor and premotor areas as when the participants actually move the effector associated with these verbs (Buccino et al., 2001; Hauk et al., 2004). This “somatotopy” is one of the major argument supporting the idea that concrete concepts are grounded in action–perception systems of the brain (Pulvermüller, 2005; Barsalou, 2008). Transcranial magnetic stimulation (TMS) results confirmed the somatotopy in human primary motor cortex (M1) demonstrating that the stimulation of the arms or legs M1 regions facilitated the recognition of action verbs involving movement of the respective extremities (Pulvermüller, 2005; Innocenti et al., 2014).

However, one of the major criticism to the embodied theory is the idea that motor system plays an epiphenomenal role during language processing (Mahon and Caramazza, 2008). In this view, the activations of motor system are not necessary to language understanding but they are the result of a cascade of spreading activations caused by the amodal semantic representation, or a consequence of explicit perceptual or motor imagery induced by the semantic tasks.

To address this point, further neurophysiological studies using time-resolved techniques such as high-density electroencephalography (EEG) or magnetoencefalography (MEG) indicated that the motor system is involved in an early time window corresponding to lexical-semantic access (Pulvermüller, 2005; Hauk et al., 2008; Dalla Volta et al., 2014; Mollo et al., 2016), supporting a causal relationship between motor cortex activation and action verb comprehension. Interestingly, recent evidences (Dalla Volta et al., 2018; García et al., 2019) has dissociated the contribution of motor system during early semantic access from the activation of lateral temporal-occipital areas in deeper semantic processing (e.g., categorization tasks) and multimodal reactivation.

Another outstanding question is raised by the controversial data about the processing of non-action language (i.e., “abstract” concepts). According to the Dual Coding Theory (Paivio, 1991), concrete words are represented in both linguistic and sensorimotor-based systems, while abstract words would be represented only in the linguistic one. Neuroimaging studies support this idea showing that the processing of abstract words is associated with higher activations in the left IFG and the superior temporal cortex (Binder et al., 2005, 2009; Wang et al., 2010), areas commonly involved in linguistic processing. The Context Availability Hypothesis instead argues that abstract concepts have increased contextual ambiguity compared to concrete concepts (Schwanenflugel et al., 1988). While concrete words would have direct relations with the objects or actions they refer to, abstract words can present multiple meanings and they needed more time to be understood (Dalla Volta et al., 2014, 2018; Buccino et al., 2019). This assumes that, they can be disambiguated if inserted in a “concrete context” which provides elements to narrow their meanings (Glenberg et al., 2008; Boulenger et al., 2009; Scorolli et al., 2011, 2012; Sakreida et al., 2013). Researches on action metaphors (e.g., “grasp an idea”) that are involved in both action and thinking, found an engagement of sensory-motor systems even when action language is figurative (Boulenger et al., 2009, 2012; Cuccio et al., 2014). Nevertheless, some studies observe motor activation only for literal, but not idiomatic sentences (Aziz-Zadeh et al., 2006; Raposo et al., 2009).

In a recent TMS study, De Marco et al. (2018) tested the effect of context in modulating motor cortex excitability during abstract words semantic processing. The presentation of a congruent manual symbolic gesture as prime stimulus increased hand M1 excitability in the earlier phase of semantic processing and speeded word comprehension. These results confirmed that the semantic access to abstract concepts may be mediated by sensorimotor areas when the latter are grounded in a familiar motor context.

## Gestures: a Bridge Between Language and Action

One of the major contribution in support of embodied cognition theory derived from the hypothesis of the motor origin of spoken language. Comparative neuroanatomical and neurophysiological studies sustain that F5 area in macaques is cytoarchitectonically comparable to Brodmann area 44 in the human brain (IFG), which is part of Broca’s area (Petrides et al., 2005, 2012). This area would be active not only in human action observation but also in language understanding (Fadiga et al., 1995, 2005; Pulvermüller et al., 2003), transforming heard phonemes in the corresponding motor representations of the same sound (Fadiga et al., 2002; Gentilucci et al., 2006). In this way, similarly to what happen during action comprehension, the MM would directly link the sender and the receiver of a message (manual or vocal) in a communicative context. For this reason, it was hypothesized to be the ancestor system favoring the evolution of language (Rizzolatti and Arbib, 1998).

Gentilucci and Corballis (2006) showed numerous empirical evidence that support the importance of the motor system in the origin of language. Specifically, the execution/observation of a grasp with the hand would activate a command to grasp with the mouth and vice-versa (Gentilucci et al., 2001, 2004, 2012; Gentilucci, 2003; De Stefani et al., 2013a). On the basis of these results the authors proposed that language evolved from arm postures that were progressively integrated with mouth articulation postures by mean of a double hand–mouth command system (Gentilucci and Corballis, 2006). At some point of the evolutionary development the simple vocalizations and gestures inherited from our primate ancestors gave origin to a sophisticated system of language for interacting with others conspecifics (Rizzolatti and Arbib, 1998; Arbib, 2003, 2005; Gentilucci and Corballis, 2006; Armstrong and Wilcox, 2007; Fogassi and Ferrari, 2007; Corballis, 2010), where manual postures became associated to sounds.

Nowadays, during a face-to-face conversation, spoken language and communicative motor acts operate together in a synchronized way. The majority of gestures are produced in association with speech: in this way the message assumes a specific meaning. Nevertheless, a particular type of gesture, the symbolic gesture (i.e., OK or STOP), can be delivered in utter silence because it replaces the formalized, linguistic component of the expression present in speech (Kendon, 1982, 1988, 2004). A process of conventionalization (Burling, 1999) is responsible for transforming meaningless hand movements that accompany verbal communication (i.e., gesticulations, McNeill, 1992) into symbolic gestures, as well as string of letters may be transformed into a meaningful word. Symbolic gestures therefore represent the conjunction point between manual actions and spoken language (Andric and Small, 2012; Andric et al., 2013). This leads to a great interest around the study of the interaction between symbolic gestures and speech, with the aim to shed light to the complex question about the role of the sensory-motor system in language comprehension.

A large amount of researches have claimed that, during language production and comprehension, gesture and spoken language are tightly connected (Gunter and Bach, 2004; Bernardis and Gentilucci, 2006; Gentilucci et al., 2006; Gentilucci and Dalla Volta, 2008; Campione et al., 2014; De Marco et al., 2015, 2018), suggesting that the neural systems for language understanding and action production are closely interactive (Andric et al., 2013).

In line with the embodiment view of language, *the theory of integrated communication systems* (McNeill, 1992, 2000; Kita, 2000) is centered on the idea that gestures and spoken language comprehension and production are managed by a unique control system. Thus, gestures and spoken language are both represented in the motor domain and they necessarily interact with each other during their processing and production.

At the opposite, the *theory of independent communication systems* (Krauss and Hadar, 1999; Barrett et al., 2005) claims that gestures and speech can work separately and are not necessarily integrated each other. Communication with gestures is described as an auxiliary system, evolved in parallel to language, that can be used when the primary system (language) is difficult to use or not intact. In this view, gesture-speech interplay is regarded as a semantic integration of amodal representations, taking place only after processing of the verbal and gestural messages have occurred separately. This hypothesis is primary supported by neuropsychological cases which reported that abnormal skilled learned purposive movements (limb apraxia) and language disorders (aphasia) are anatomically and functionally dissociable (Kertesz et al., 1984; Papagno et al., 1993; Heilman and Rothi, 2003). However, limb apraxia often co-occuring with Broca’s Aphasia (Albert et al., 2013) and difficulty in gesture-speech semantic integration was reported in aphasic patients (Cocks et al., 2009, 2018). Alongside clinical data, disrupting the activity in both left IFG and middle temporal gyrus (MTG) is found to impair gesture-speech integration (Zhao et al., 2018).

Evidence in favor of the integrated system theory came from a series of behavioral and neurophysiological studies that have investigated the functional relationship between gestures and spoken language. The first evidence of the reciprocal influence of gestures and words during their production came from the study by Bernardis and Gentilucci (2006), who showed how the vocal spectra measured during the pronunciation of one word (i.e., “hello”) was modified by the simultaneous production of the corresponding in meaning gesture (and vice-versa, the kinematics resulted inhibited). This interaction was found depending on the semantic relationship conveyed by the two stimuli (Barbieri et al., 2009), and was replicated even when gestures and words were simply observed or presented in succession (Vainiger et al., 2014; De Marco et al., 2015).

Neurophysiological studies showed controversial evidences about the core brain areas involved in gestures and words integration, that include different neural substrates as M1 (De Marco et al., 2015, 2018) IFG, MTG and superior temporal gyrus/sulcus (STG/S) (Willems and Hagoort, 2007; Straube et al., 2012; Dick et al., 2014; Özyürek, 2014; Fabbri-Destro et al., 2015). However, IFG virtual lesion showed to disrupt gesture-speech integration effect (Gentilucci et al., 2006), in accordance with the idea of human Broca’s area (and so the mirror circuit) as the core neural substrate of action, gesture and language processing and interplay (Arbib, 2005). Partially in contrast, investigation of temporal dynamics of the integration processing by mean of combined EEG/fMRI techniques confirmed the activation of a left fronto-posterior-temporal network, but revealed a primary involvement of temporal areas (He et al., 2018).

Finally, further results in favor of motor origin of language came from genetic research, since it was suggested that FOXP2 gene was involved both in verbal language production and upper limb movements coordination (Teramitsu et al., 2004) opening the question about a possible molecular substrate linking speech with gesture (see Vicario, 2013).

In conclusion, a good amount of results evidenced a reciprocal influence between gesture and speech during their comprehension and production, showing overlapping activation of the MM neural systems (IFG) involved in action, gesture and language processing and interplay (see Table 1). Further studies should consider potential integration of neuroscience research with promising fields investigating the issue at molecular level.

**TABLE 1:** Summary of main concepts, neural evidence, and future challenges about the theories explaining language semantic processing and evolution.

	**Semantic processing**	
	
	**Embodied theory**	**Amodal/Symbolic theory**
Main concepts	Conceptual knowledge is grounded in body experience and in the sensorimotor systems	Semantic concepts are stored and processed as formal symbols
Neural systems	Primary motor and sensory systems, Fronto-Parietal Mirror Circuit	Temporal cortex (Anterior Temporal Lobe, Middle Temporal Gyrus)
Main references	Gallese and Lakoff, 2005; Barsalou, 2008; Casile, 2012; Kiefer and Pulvermüller, 2012	Fodor, 1998; Patterson et al., 2007; Mahon and Caramazza, 2009; Visser et al., 2010
Challenges	No shared model about the dynamic and interplay between sensorimotor and temporal brain areas at different stages of semantic comprehension Necessity to further support the essential contribute of sensorimotor system in abstract language processing	

	**Language evolution**	
	
	**Gestural origin of Language**	**Independent evolution of gestures and language**

Main concepts	Speech evolved from arm postures that were progressively integrated with mouth gestures and vocalization by mean of a double hand–mouth command system. Gesture and speech necessarily interact during their processing and production	Gestures and speech evolved independently. They are functionally dissociated and processed separately, or eventually integrated as amodal concepts). Communication with gestures is described as an auxiliary system
Neural systems	Inferior Frontal Gyrus	Sensorimotor systems for gestures, temporal cortex for language
Main references	McNeill, 1992; Rizzolatti and Arbib, 1998; Gentilucci and Corballis, 2006; Gentilucci et al., 2006	Krauss and Hadar, 1999; Barrett et al., 2005
Challenges	Overlapping activation of areas belonging to mirror circuit (IFG) and linguistic areas (MTG) during gesture and speech processing Limited evidence about neural dynamic of gesture and speech interplay Potential fields of research (i.e., FOXP2 genes variations and communication behavior)	

## Motor Signs in Emotional Communication

The majority of studies that investigated the neural mechanism of hand gesture processing focused on the overlapping activations of words and gestures during their semantic comprehension and integration. However, it was shown that, gestural stimuli can convey more than semantic information, since they can also express emotional message. A first example came from the study of Shaver et al. (1987) which tried to identify behavioral prototype related to emotions (e.g., fist clenching is involved in the anger prototype). More recently, Givens (2008) showed that uplifted palms postures suggest a vulnerable or non-aggressive pose toward a conspecific.

However, beyond hand gestures investigations, emerging research about the role of motor system in emotion perception dealt with the study of mechanisms underlying body postures and facial gestures perception (De Gelder, 2006; Niedenthal, 2007; Halberstadt et al., 2009; Calbi et al., 2017). Of note, specific connections with limbic circuit were found for mouth MNs (Ferrari et al., 2017), evidencing the existence of a distinct pathway linked to the mouth/face motor control and communication/emotions encoding system. These neural evidences are in favor of a role of MM in the evolution and processing of emotional communication through the mouth/facial postures. As actions, gestures and language become messages that are understood by an observer without any cognitive mediation, the observation of a facial expression (such as disgust) would be immediately understood because it evokes the same representation in the insula of the individual observing it (Wicker et al., 2003).

We propose that MM guides every-day interactions in recognizing emotional states in others, decoding body and non-verbal signals together with language, influencing and integrating the communicative content in the complexity of a social interaction.

Indeed, the exposure to congruent facial expressions was found to affect the recognition of hand gestures (Vicario and Newman, 2013), as the observation of facial gesture interferes with the production of a mouth posture involving the same muscles (Tramacere et al., 2018).

Moreover, emotional speech (prosody), facial expressions and hand postures were found to directly influence motor behavior during social interactions (Innocenti et al., 2012; De Stefani et al., 2013b, 2016; Di Cesare et al., 2017).

## Conclusion and Future Directions

Numerous behavioral and neurophysiological evidences are in favor of a crucial role of MM in language origin, as in decoding semantic and emotional aspects of communication.

However, some aspects need to be further investigated, and controversial results were found about the neural systems involved in semantics processing (especially for abstract language).

Nevertheless, a limitation emerges about experimental protocols which studied language in isolation, without considering the complexity of social communication. In other words, language should be considered always in relation to some backgrounds of a person mood, emotions, actions and events from which the things we are saying derive their meanings. Future studies should adopt a more ecological approach implementing research protocols that study language in association to congruent or incongruent non-verbal signals.

This will shed further light onto the differential roles that brain areas play and their domain specificity in understanding language and non-verbal signals as multiple channels of communication.

Furthermore, future research should consider to integrate behavioral and combined neurophysiological technique extending the sampling from typical to psychiatric population.

Indeed, new results will have also important implications for the comprehension of mental illness that were characterized by communication disorders and MM dysfunction as Autism Spectrum Disorder (Oberman et al., 2008; Gizzonio et al., 2015), schizophrenia (Sestito et al., 2013), and mood disorders (Yuan and Hoff, 2008).

## Author Contributions

All authors listed have made a substantial, direct and intellectual contribution to the work, and approved it for publication.

## Conflict of Interest Statement

The authors declare that the research was conducted in the absence of any commercial or financial relationships that could be construed as a potential conflict of interest.
